# The way out: TPT3 allows triose-P export from the chloroplast

**DOI:** 10.1093/plcell/koad106

**Published:** 2023-04-13

**Authors:** Solène L Y Moulin

**Affiliations:** Assistant Features Editor, The Plant Cell, American Society of Plant Biologists; Department of Pathology, Stanford University School of Medicine, Stanford, CA 94305, USA

Just as rivers can overflow and bring devastating floods during heavy rainfall, high light intensity can flood the photosynthetic machinery with damaging free radicals. To cope with changing light intensities, plants rely on several mechanisms acting both upstream and downstream of photosynthesis. These include dissipating absorbed excitation energy upstream of the photosynthetic reaction centers and exporting downstream photosynthetic products such as reductants and organic carbon produced by the Calvin-Benson-Bassham (CBB) cycle from the chloroplast. Metabolic flux analysis during heterotrophic growth of the model green alga *Chlamydomonas reinhardtii* (Chlamydomonas) suggested that the activity of triose phosphate/inorganic phosphate translocators (TPTs) exceeds that of the malate-OAA reductant shuttle, thereby allowing the export of organic carbon while maintaining chloroplast phosphate homeostasis (Boyle et al. 2017). However, TPTs have not been studied in detail in Chlamydomonas. Now, Huang et al. (2023) have used a reverse genetics approach to investigate the presence and function of these organic carbon transporters in the Chlamydomonas chloroplast envelope.

The presence of TPTs in Chlamydomonas was investigated based on homology analysis with Arabidopsis TPTs. Of all TPT candidates, TPT2, TPT3, TPT10, and CGL51 contained a predicted chloroplast transit peptide. TPT2 and TPT3 were selected for further analysis based on their similarities with plant TPTs (Bockwoldt et al. 2019) and high expression levels. TPT2 and TPT3 protein fusions to VENUS confirmed their localization to the chloroplast envelope. Heterologous expression of *TPT2* and *TPT3* in budding yeast allowed the reconstitution of translocator-containing liposomes. Subsequent liposome assays for the rate of triose phosphate/Pi exchange showed that both translocators have a typical TPT substrate spectrum and a similar substrate preference for dihydroxyacetone phosphate and 3-phosphoglycerate.

To further investigate the function of these chloroplastic TPTs, *tpt2* and *tpt3* knockouts were generated using CRISPR/Cas9. Under photoautotrophic and heterotrophic conditions, *tpt2* growth was inhibited only by high light, whereas *tpt3* grew poorly even in low light and could not grow under moderate or high-light conditions. Lugol and Nile red staining revealed high levels of starch and lipid accumulation in *tpt3* mutants but not in the wild-type cells following exposure to high light. These results suggest that TPT3 is important for the export of energy/fixed carbon from the chloroplast, particularly under high-light conditions. Furthermore, whereas other sugar transporters seemed to be able to partially compensate for the loss of TPT2, there appeared to be little compensation for the loss of TPT3. The *tpt3* mutant phenotype was unexpectedly severe compared with known mutants in higher plants where transporters mostly compensate one another.

A comparative metabolite analysis showed that the *tpt3* mutant contained a larger pool of glycolytic/gluconeogenic/CBB cycle intermediates but similar or lower levels of TCA and glyoxylate cycle intermediates compared with the wild-type cells. These results confirmed the function of TPT3 in the export of photosynthetic products and support the hypothesis that triose-P export drives the cytosolic segment of glycolysis and downstream metabolic pathways. Moreover, these findings confirm that the flux of fixed carbon as hexose-P from the chloroplast is negligible in Chlamydomonas (Treves et al. 2022). The light sensitivity of *tpt3* was related to photosystem II damage, as revealed by a reduced oxygen evolution rate and photosystem II yield. In addition, *tpt3* showed hyper-reduction of the photosynthetic electron transport chain, a higher plastoquinone pool reduction state, and a slower rate of photosystem I oxidation. Additionally, with increasing light intensity, *tpt3* accumulated higher levels of reactive oxygen species in the stroma, cytosol, and nucleus than the wild-type cells. Surprisingly, the reactive oxygen species levels were lower in mitochondria, which might be due to lower activity of the mitochondrial electron transport chain as a consequence of lower carbon export from the chloroplast. All these observations are consistent with a bottleneck in the export of CBB cycle products from the chloroplast in *tpt3*, which causes saturation of the chloroplast photosynthetic capacity, even in moderately low-light conditions (see Fig.).

**Figure.**
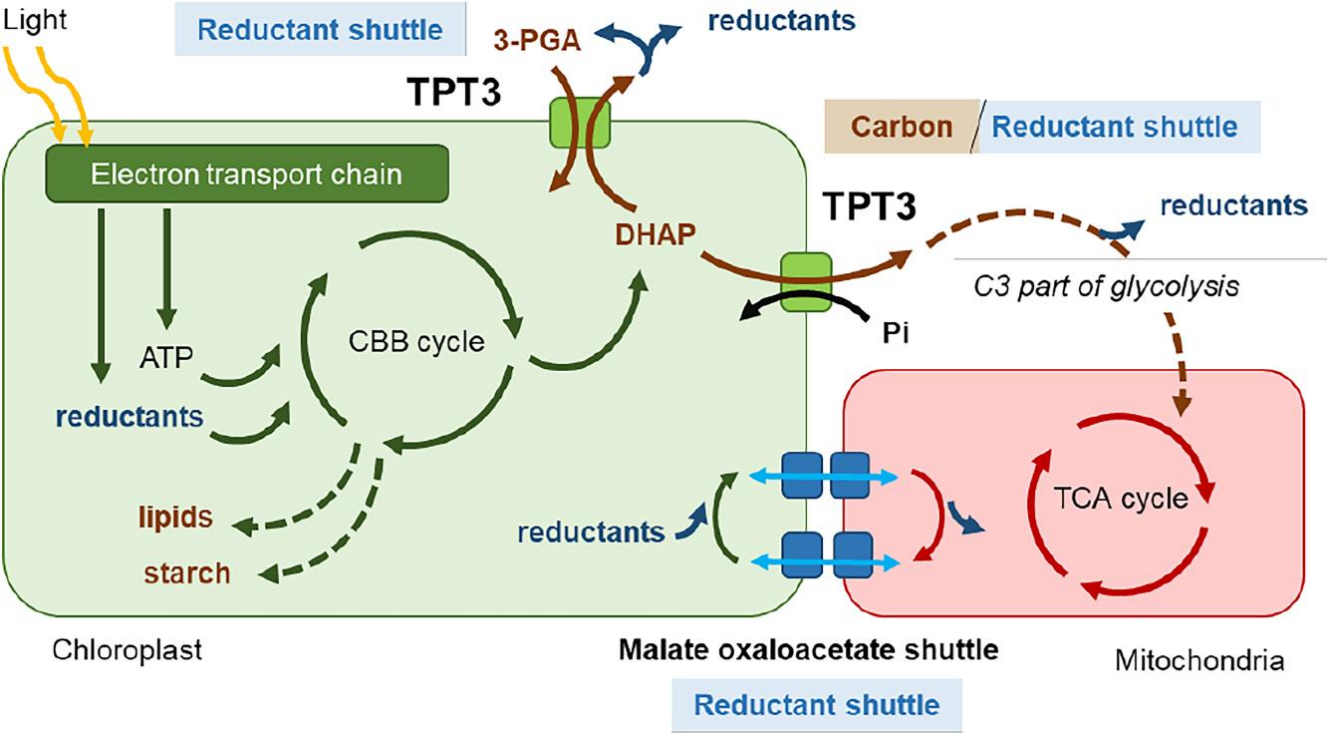
Role of Chlamydomonas TPT3 in the cellular metabolic landscape. TPT3 allows the export of reductants and organic carbons produced by photosynthesis from the chloroplast. TPT3 links chloroplast metabolism with cytoplasmic and mitochondrial metabolism. Figure credit: S. Moulin, adapted from Huang et al. (2023), Figure 7.

This study highlights the importance of TPTs in carbon and energy export from chloroplasts and provides new insights into the metabolic pathways that drive the growth and adaptation of microalgae in response to different light intensities. Understanding these pathways creates opportunities to tailor microalgae for the biotechnological production of lipids, starch, and other bioproducts.

## References

[koad106-B1] Bockwoldt M , Heiland I, Fischer K. The evolution of the plastid phosphate translocator family. Planta. 2019:250(1):245–261. 10.1007/s00425-019-03161-y 30993402

[koad106-B2] Boyle NR , Sengupta N, Morgan JA. Metabolic flux analysis of heterotrophic growth in *Chlamydomonas reinhardtii*. PLoS One. 2017:12(5):e0177292. 10.1371/journal.pone.0177292 28542252 PMC5443493

[koad106-B3] Huang W , Krishnan A, Plett A, Meagher M, Linka N, Wang Y, Ren B, Findinier J, Redekop P, Fakhimi N, et al *Chlamydomonas* mutants lacking chloroplast TRIOSE PHOSPHATE TRANSPORTER3 are metabolically compromised and light-sensitive. Plant Cell. 2023:35(7):2592–2614. 10.1093/plcell/koad095 PMC10291034 36970811

[koad106-B4] Treves H , Küken A, Arrivault S, Ishihara H, Hoppe I, Erban A, Höhne M, Moraes TA, Kopka J, Szymanski J, et al Carbon flux through photosynthesis and central carbon metabolism show distinct patterns between algae, C3 and C4 plants. Nat Plants. 2022:8(1):78–91. 10.1038/s41477-021-01042-5 34949804 PMC8786664

